# Establishing a postgraduate programme in nutritional epidemiology to strengthen resource capacity, academic leadership and research in the democratic republic of Congo

**DOI:** 10.1186/s12909-021-02557-3

**Published:** 2021-02-27

**Authors:** Mapatano Mala Ali, Lyn Haskins, Vaughn John, Anne Hatløy, Silondile Luthuli, Sphindile Mapumulo, Ingunn M. S. Engebretsen, Thorkild Tylleskär, Paulin Mutombo, Christiane Horwood

**Affiliations:** 1 Kinshasa School of Public Health, University of Kinshasa, Kinshasa, Democratic Republic of Congo; 2 Centre for Rural Health, School of Nursing and Public Health, University of KwaZulu-Natal, Durban, South Africa; 3 School of Education, University of KwaZulu-Natal, Pietermaritzburg, South Africa; 4 Centre for International Health, University of Bergen, Bergen, Norway; 5 Fafo Institute for Labour and Social Research, Oslo, Norway

**Keywords:** Education, LMIC, Research capacity, Partnerships, North-south-south, Nutrition, Epidemiology, DRC, Africa

## Abstract

**Background:**

Low- and Middle-income countries (LMIC) face considerable health and nutrition challenges, many of which can be addressed through strong academic leadership and robust research translated into evidence-based practice. A North-South-South partnership between three universities was established to implement a master’s programme in nutritional epidemiology at the Kinshasa School of Public Health (KSPH), Democratic Republic of Congo (DRC). The partnership aimed to develop academic leadership and research capacity in the field of nutrition in the DRC. In this article we describe the educational approach and processes used, and discuss successes, challenges, and lessons learned.

**Methods:**

Self-administered questionnaires, which included both open and closed questions, were sent to all graduates and students on the master’s programme to explore students’ experiences and perceptions of all aspects of the educational programme. Quantitative data was analysed using frequencies, and a thematic approach was used to analyse responses to open-ended questions.

**Results:**

A two-year master’s programme in Nutritional Epidemiology was established in 2014, and 40 students had graduated by 2020. Key elements included using principles of authentic learning, deployment of students for an internship at a rural residential research site, and support of selected students with bursaries. Academic staff from all partner universities participated in teaching and research supervision.

The curriculum and teaching approach were well received by most students, although a number of challenges were identified. Most students reported benefits from the rural internship experience but were challenged by the isolation of the rural site, and felt unsupported by their supervisors, undermining students’ experiences and potentially the quality of the research. Financial barriers were also reported as challenges by students, even among those who received bursaries.

**Conclusion:**

The partnership was successful in establishing a Master Programme in Nutritional Epidemiology increasing the number of nutrition researchers in the DRC. This approach could be used in other LMIC settings to address health and nutrition challenges.

**Supplementary Information:**

The online version contains supplementary material available at 10.1186/s12909-021-02557-3.

## Introduction

Building research capacity in low- and Middle-income countries (LMIC) and embedding skilled researchers into health and management systems to develop evidence-based health policies is fundamental to tackle the substantial health challenges these countries face. Policies firmly based on evidence-based interventions, which are locally relevant and applicable, have the potential to improve health in LMIC and reduce health inequities [1]. The World Health Organization (WHO) has identified developing strong leadership in the global health workforce as a priority, and has established partnerships to strengthen the research infrastructure and capacity in the public health arena in African Universities [2]. This has led to the development of many collaborations between high-income countries, mainly from the global North, and LMICs in the global South, which have played a strong role in developing research capacity. However, there have been concerns that uprooting LMIC students to study in high-income countries may provide students with skills that are not relevant or applicable to their local context [3]. In addition, there may be a brain drain associated with out-of-country training, particularly for long periods, as students become rooted in the host country and fail to return. Another key challenge has been mismatches between the health needs of the South partners and research investments of the North partners, leading to partners in the North driving the research agenda, focusing research activities away from local priorities [4].

In response to these challenges, the focus of these collaborations has shifted towards more equal North-South partnerships and health research capacity building initiatives aimed at supporting demand-driven and locally led research. These partnerships have employed a number of different approaches, including training courses to build and strengthen research skills [5–9], strengthening research infrastructure [6], supporting and training doctoral students [7, 9, 10], and training master’s students who remain in their country of origin [11] or within the African continent [8]. Other approaches have successfully engaged students in international working environments either through nesting students in research projects [10], or with short term exchanges, thus providing the southern partner with a stable locus of expertise within their own country [12].

A variety of technology-based teaching and learning methodologies have recently been introduced. Blended learning, combining face-to-face learning with electronic resources and online interactions, was able to effectively draw on skills in different countries to support global health research capacity building, but set up was costly [5, 13]. An e-learning approach was successfully able to provide health professionals working in LMIC with the opportunity to obtain a master’s degree using entirely online resources on a free open online platform [11]. However, these collaborations remain challenging with unequal power relationships between partners, lack of buy-in and leadership between partners, differing time zones and partner schedules, and technology being new to partners [6].

Nutrition is an important field for health research capacity building, since malnutrition remains a leading cause of ill health globally. Addressing malnutrition is important for achieving Sustainable Development Goal (SDG) goal 2 of zero hunger, and is therefore a key priority in many low-income countries. Nutrition interventions are frequently context-specific, making this a field of study where local research expertise is particularly important to develop and evaluate affordable, acceptable, evidence-based interventions to inform policy. Nutritional epidemiology is a relatively new field of public health and concerns the patterns, causes and solutions to nutrition problems, focusing in particular on the relationship between diet and disease. Skills development in nutritional epidemiology increases capacities to address the burden of nutrition problems, including food insecurity, malnutrition, and micronutrient deficiencies across the lifespan. Few African universities offer postgraduate studies in nutritional epidemiology.

This article describes the approach taken by a North-South-South partnership, to train master’s and doctoral students in nutritional epidemiology, at Kinshasa School of Public Health (KSPH), University of Kinshasa (UNIKIN), Democratic Republic of Congo (DRC). The DRC has some of the highest rates of undernutrition globally, and a poor track record in health research [9, 14, 15]. In this paper we focus on the master’s programme, describing key concepts underpinning its development and implementation, and describe successes and challenges from the perspectives of the master’s students.

## Description of the nutritional epidemiology master’s programme

The ‘Growing Partnership for Higher Education and Research in Nutritional Epidemiology in DRC’ (GROWNUT) project was a partnership between Centre for International Health (CIH), University of Bergen (Norway), Centre for Rural Health (CRH), University of KwaZulu-Natal (South Africa), and KSPH, UNIKIN (DRC). GROWNUT was established in 2014, in partnership with the DRC National Nutrition Programme (PRONANUT) at the DRC Ministry of Health, to establish a masters programme in nutritional epidemiology at KSPH. The vision of the partnership was to develop a cadre of skilled researchers in DRC with competencies to undertake quality nutrition research to inform evidence-based policies and practices. The partnership was funded by the Norwegian Agency for Development Cooperation through the Norwegian Programme for Capacity Development in Higher Education and Research for Development (NORHED).

### Project setting

The DRC is a low-income country [16] with a population in excess of 84 million people [17]. There is widespread food insecurity in DRC, and more than 2 million children suffer from acute malnutrition and 43% of children are stunted [18]. Infant mortality of 68 per 1000 live births and under 5 mortality of 91 per 1000 live births are among the highest in the world [19].

Higher education is provided in universities, polytechnics or specialized colleges. A number of public and private universities in the DRC established since the 1950’s, offer a wide variety of fields of study at undergraduate and post-graduate levels. Situated within UNIKIN, the KSPH offered four master’s degree programmes. However, there were no courses in nutritional epidemiology in the DRC.

### Key elements of the master Programme in nutritional epidemiology

KSPH typically offers a one-year master’s programme. However, for the nutritional epidemiology programme the period of study was extended to two years in line with master’s programmes at partner universities. The two-year programme provided students authentic learning opportunities to develop higher learning thinking, depth of knowledge, real-world problem-solving skills and student directed learning [20, 21] in the field of nutritional epidemiology.

### Curriculum

Using a constructivist approach, principles of authentic learning framed the development of the curriculum for the master’s programme in nutritional epidemiology, which included theory modules delivered in the first year, and a practical internship at a rural site during the second year of study.

The curriculum content combined concepts of nutrition, epidemiology, research methods and data management, while accounting for global issues such as climate change, natural resource management and food security. The theory component consisted of 17 modules, which were co-facilitated by international and local facilitators, bringing together the skills and perspectives of partner institutions. The aim was that the nutritional epidemiology teaching responsibilities would be handed over to local facilitators at KSPH, so ensure sustainability of the programme. However, due to political unrest limiting travel to the DRC, international facilitators were unable to co-facilitate after the second cohort. Students from the third and fourth cohorts travelled to South Africa to attend a module on research proposal development with facilitators from all partner institutions.

Teaching methods employed an adult learning approach focusing more on the learning process and less on the content of the teaching. Working in groups and as individuals, students were actively involved in their own learning, often having to research a particular topic under discussion, and present it in the classroom setting where it was critiqued and discussed by other students and facilitators. Audio-visual presentations brought global challenges to the classroom setting where these were discussed between students and facilitators with expertise in the field.

### Rural internship

A residential rural research site, with accommodation and study facilities, was established in Popokabaka health zone, in Kwango province, approximately 380 km south-east of Kinshasa. Popokabaka is an area with particularly high rates of malnutrition [22]. Students were deployed in the rural research site for 3-months practical internship and an additional 1-month to collect data for their research projects. The rural internship aimed to build on the theoretical component, with a ‘learning by doing’ practical component where students observed nutrition conditions first hand in a rural community. Students gained experience of interacting with communities and staff in local health facilities. Facilitators from KSPH regularly visited the students in the rural site to provide support and interactive teaching. During their internship, students gained skills in nutritional assessment, and collected research data.

### Students’ research projects

Individual research projects, based on research questions identified by students, formed an important component of the programme. Two supervisors were allocated to each student, the main supervisor at KSPH and a co-supervisor from a collaborating university, bringing different perspectives to the students’ research. Students were guided through proposal writing, collecting and analysing data, and writing a dissertation. Students defended their dissertation with key leaders in their field present prior to the completion of their degree. UNIKIN conferred the Master in Nutritional Epidemiology when all academic requirements had been met.

### Medium language of teaching and learning

English was adopted as a medium of instruction at the request of the DRC partner, because of perceptions that most nutrition-related research was published in English, and because English was the common language among partners and funders. Employing English as the medium of instruction aimed to strengthen the students’ ability to read and contribute to the scientific literature, participate in the discourse in the nutrition field, and set up collaborative networks outside the DRC. Therefore, students enrolling for the Master in Nutritional Epidemiology needed a working knowledge of English, and applicants undertook an assessment examination, that included an English assessment. As French was the medium of instruction for basic schooling and undergraduate teaching in DRC, this may have reduced the number of students applying to the programme.

### Provision of bursaries

Bursaries were provided for selected students; five bursaries were allocated for each cohort, including one to an existing KSPH staff member for internal capacity building. The remaining four bursaries were allocated to vulnerable groups such as students from war-torn areas and female students.

### Achievements of the master’s programme

Forty-one students registered for the Master in Nutritional Epidemiology over four cohorts, (Table 1). Three master’s students were employed by KSPH, one of whom is currently completing PhD studies supported by the project.

**Table 1:** Students participating in the nutritional epidemiology master’s programme by cohort

	Year of study	Number of students graduated
Cohort 1	2014–2016	10
Cohort 2	2015–2017	11 (1 failed)
Cohort 3	2016–2018	12
Cohort 4	2017–2019	7

Forty students graduated with a Master in Nutritional Epidemiology, of whom 34 wrote their thesis in English and six wrote in French. Research topics spanned a variety of nutritional challenges in DRC and most students (35) collected data at the rural research site. Examples of research topics are shown in Table 2.

**Table 2:** Examples of research undertaken by Nutritional Epidemiology students

Double burden of malnutrition among adolescents in Popokabaka	
Positive deviance as an approach for preventing acute malnutrition in rural context of Popokabaka	
Evaluation of the Double Burden of Malnutrition: Under nutrition & overweight / obesity in children under five years in the city of Popokabaka in Kwango (DRC)	
Breastfeeding practices among lactating women living in the Health Area of Popokabaka, Kwango Province, DRC	
Dietary knowledge and practices during pregnancy among pregnant women and key informants in the rural area of Popokabaka	
Exploration of nutrition counselling provided to pulmonary TB patients in Popokabaka Health Zone	
Exploration of nutritional counselling for people living with HIV followed up at Popokabaka hospital	
Relationship between women’s education level and infant feeding practices in Popokabaka health district	
Exploring the perception of the Popokabaka population about the choice of sources of drinking water	
Barriers to exclusive breastfeeding practices among mothers with children aged 0–6 months in Popokabaka, Kwango Province, DRC	
Care-seeking behaviors of mothers/caregivers about acute malnutrition in children under 5 years of age in Popokabaka	
Food insecurity as a barrier to antiretroviral therapy in Popokabaka	
Knowledge and feeding practices during pregnancy among mothers who delivered a low birth weight infant in the Popokabaka Health Zone	
The relationship between breakfast consumption and school performance among school aged children in Lemba municipality	

Eleven master’s students presented research findings at international conferences and five manuscripts to be submitted in academic recognised journals are in preparation by master’s students, supported by project staff. As a result of networking at a conference, one master’s graduate set up the “Societé Congolaise de Nutrition et d’Alimentation” (SCONUTAL) society, networking with the SUN-DRC movement, a civil organisation to advocate for investment in national food and nutrition programmes and promote research in the field of nutrition.

In addition, a mini- conference was held at the rural research site in February 2019 to provide feedback about research findings to the local community, including representatives from the Health Zone and Administrative Territory, religious representatives, local NGOs, and stakeholders. PhD and master’s students and KSPH academic staff presented findings.

On completion of their degree, master’s graduates have been employed in a variety of nutrition specific fields such as PRONANUT (4), WHO (1), UNICEF (2) and other non-governmental organisations (5) where they are able to represent the DRC in the international arena. Others are employed in teaching capacities, not only at the University of Kinshasa (3), but also other universities and training colleges (2). Two graduates have returned to work in Popokabaka, while others work in hospitals, and rural health zones. One student registered for a PhD at UNIKIN and two students have registered for PhDs at other universities.

## Students’ evaluation of the master degree in nutritional epidemiology

## Methods

### Study design

A cross-sectional descriptive study was conducted to evaluate the master’s programme in nutritional epidemiology from the perspective of students.

### Participants

Forty students who enrolled in the Master Programme in Nutritional Epidemiology between 2014 and 2018 were invited to participate in the survey, including 32 graduates and eight currently enrolled students. All participants had completed the learning components of the programme. One student who failed the theory modules was excluded from the evaluation.

### Data collection tools

**Fig. 1:**
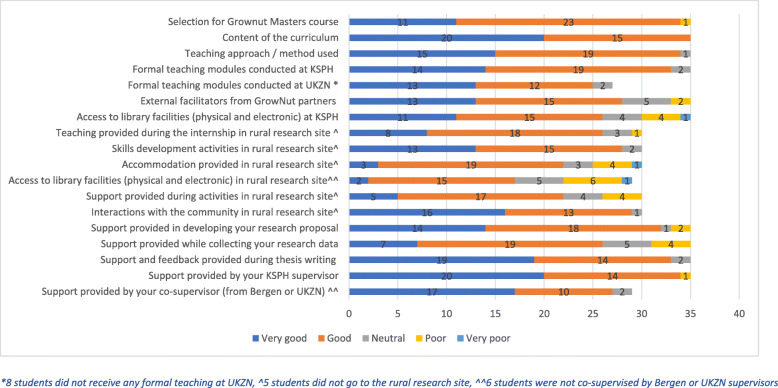
Students rating of the activities undertaken in the Master Programme in Nutritional Epidemiology

**Fig. 2:**
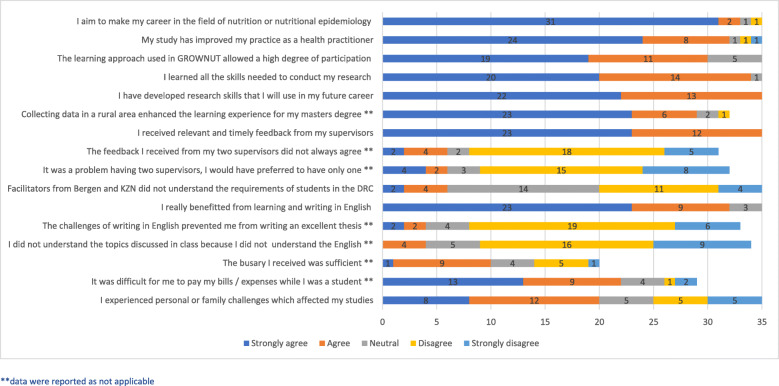
Students experiences of the Master Programme in Nutritional Epidemiology

A quantitative questionnaire was developed with both closed and open-ended questions. Participants were requested to rate the quality of all elements of the master’s programme using Likert-type scales (Fig. 1), and asked to agree or disagree with a series of statements relating to the quality of the master’s programme (Fig. 2). Open ended questions allowed students to comment in their own words about what they valued most or least about the different components of the master’s programme. Questionnaires were developed in English and translated into French. Students were requested to respond in the language of their choice.

### Data collection and management

Self-administered questionnaires were emailed to all 40 students requesting them to participate, and emails were re-sent to remind non-responders to participate on five occasions over a four-month period. Questionnaires used an online platform which students downloaded, completed and then returned via email. One staff member from UKZN who had not been involved in the GROWNUT project, received the completed questionnaires, anonymized them by allocating a study number and removing identifying information.

### Data analysis

All questionnaires were double entered and validated in Excel 2019 and converted to SPSS V26 for analysis. Descriptive statistics are presented. Frequencies for rating of the master’s programme and levels of agreement and disagreement with specific statements are shown in aggregated stacked bars. Where respondents reported a question as not applicable, the denominator was changed to reflect this. Open ended questions were categorized into themes and verbatim quotes used to highlight specific points and opinions.

## Results

Data were collected between November 2019 and February 2020. In total 35/40 (88%) students responded to the questionnaire including respondents from each of the four cohorts.

Characteristics of respondents are shown in Table 3.

**Table 3:** Characteristics of participants

	*n* = 35
Age of participants (median)	39 years
	IQR 32–44 years
**Gender**	
Male	23
Female	12
**Profession / Basic degree**	
Doctor / Medicine / Physician	24
Nutritionist	3
Other	2
Missing data	6
**Cohort of GROWNUT students**	
1st cohort	7
2nd cohort	9
3rd cohort	9
4th cohort	8
Missing data	2
**Student participated in an internship in rural research site**	
Yes	30
No	5
**Student collected data for thesis in rural research site**	
Yes	26
No	9
**Degree is completed**	
Yes	32
No	3
**Student received a GROWNUT bursary**	
Yes	15
No	12
Not applicable / missing data	8
**Language in which thesis was written**	
English	29
French	6

Most aspects of the Master Degree in Nutritional Epidemiology were rated as either good or very good. Aspects given a poor or very poor rating by a minority of the students were: the selection process for admission to the programme; external facilitators from GROWNUT partners; support provided during the rural internship; accommodation at the rural site; support for proposal development; support provided during data collection. (Fig. 1).

Agreement or disagreement with statements indicated how students experienced important aspects of the master’s programme including how participating affected them personally (Fig. 2).

### English medium of instruction

Most students (23/35) strongly agreed they benefitted from learning in English but a few remained neutral (3/35). While no student disagreed they had benefitted from learning in English, a small number of students (4/35) expressed that challenges of writing their thesis in English resulted in their being unable to submit an excellent thesis. Similarly, a few students (4/35) responded that they were unable to understand some topics discussed in the classroom because they did not understand English (Fig. 2).

### Financial/personal challenges

Twenty two of the 35 students indicated they had encountered difficulties paying their bills or expenses while they were a student, and 20/35 students reported experiencing personal challenges while studying. Among 20 students who had received a bursary, 10 reported that the bursary was sufficient for their needs.

### Research skills development

Collecting data in the rural research site was described as contributing towards a positive learning experience by most students (29/35). While all 35 students reported they had developed research skills that would benefit their future, and that supervisors provided timely feedback (35/35), there were a few (5/35) who communicated they had experienced challenges in coping with two supervisors for their research and would have preferred to only have one supervisor.

### Responses to open-ended questions

Students used open-ended questions to describe the best and worst aspects of programme in their own words. Teaching methods used in the GROWNUT programme, learning in English, and experience gained at the rural research site were among the best aspects reported by students. Feeling isolated during the rural internship and financial difficulties were reported as worst aspects. In addition, students gave a number of recommendations about how the programme could be improved (Table 4).

**Table 4:** Students perceptions of the best and worst aspects of the Master Programme in Nutritional Epidemiology (open questions)

BEST things about GrowNut programme	WORST things about GrowNut programme	Students recommendations
**Curriculum**		
*The content of the theoretical courses* (GN10) *Epidemiology and Statistics courses were much more advanced, compared to other Master options* (GN16) *Bio statistics and epidemiology* (GN23) *Learning data entry and analysis software* (GN24) *Course contents were well designed*. (GN33) *Richness of the subject matter* (GN34)	*The qualitative research course was botched (GN11)* *Qualitative research module was not well provided (GN17)*	*To add a behavioural communication module into the program, this will help prevent malnutrition at the community level (GN07)* *Deepen the qualitative research course (GN11)* *A health communication course would be of great importance for the contact between the community and the researchers (GN13)* *If we can add the courses in follow-up evaluation, project management and scientific communication (GN23)* *Introduce a communication course in the programme and the development of emergency projects (GN24)*
**Teaching methods**		
*Interactive teaching and to do homework in groups* (GN04) *Facilitation of courses by teachers from the universities of Bergen and KZN* (GN05) *Benefit from teaching with rigorous foreign teachers in their supervision.* (GN09) *Communication with certain facilitators from different universities* (GN11) *Experiences of the different local and expatriate professors / facilitators* (GN12) *Being taught by professors from other countries university* (GN15) *Knowledge sharing from Bergen /UKZN/DRC facilitators (GN22)* *Professors from “3 reality of the world” (DRC, South Africa and Bergen /Norway). (GN27)*	*Concentration of [too many] courses in one year.* (GN33) *Studied for 2 years but transcripts are only for one-year Master* (GN16)	*Reduce the training period to 18 months (GN05)* *To create openings within the framework of GrowNut to continue my doctoral studies in epidemiology (GN08)* *Increase the number of hours of field practice during the 1st year of internship (GN11)* *There are modules that can be given in a conference setting (GN14)* *Respect the time set for training (length of training). Training takes 27 months or more instead of 24 as planned (GN18)* *Recommend that theoretical courses are given in 2 years instead of doing it in one year (GN31)*
***English language as a medium of instruction***		
*Learning courses and writing a thesis in English (GN03)* *The fact of writing my thesis in English (GN09)* *English. I have learnt new technical vocabulary, new words and new expressions (GN16)* *Learning the lessons of the second block which was done entirely in English (GN17)* *Different courses seen in practice and improved my English. (GN21)* *Learning an important part of our study in English (GN26)* *The course of high standards in English (GN27)* *I gave myself a discipline to have extra classes in English. (GN 35)*	*Requirement to write, to be evaluated, to receive such relevant lessons and a thesis in a language that I do not master [English] (GN06)* *Poor English language skills have delayed some things (GN24)*	*Introduce … the English course 6 months before the start of the courses for capacity building (GN24)*
***Bursaries***		
*Obtaining the scholarship, taking charge of my delivery, the payment of the child’s nanny. (GN13)* *Obtaining my scholarship (GN15)* *Opportunity to get a scholarship. (GN20)*	*Difficult to pay my bills while I was studying (GN20)* *The criteria set up to give the bursary for the PHD degree (GN27)* *Not to have benefited from the scholarship (GN30)* *Difficulties to pay academic fees for both years, without scholarship (GN35)*	*Fair bursary distribution is required (GN04)* *Increase the number of scholarships (GN05)* *provide life insurance / health insurance (GN11)* *Provide pocket money for scholarship learners (GN18)* *include in the bursary [selection] people who are not [resident] in the capital (GN29)* *Feed external students, even with a meal, to increase their attention [span] during lessons (GN33)*
***The rural research site – internships***		
*Internship in Popokabaka. (GN01)* *Participate in internship in Popokabaka. (GN04)* *Field work (training) in rural environment (GN07)* *Internship spent at Popokabaka, which allowed the learners to contextualize the theoretical lessons in the field. (GN08)* *New experience working in the bush (on the field) (GN15)* *The internship of Popokabaka (GN17)* *Practical course carried out in Popokabaka which refined all the theory learned for 2 years. (GN30)*	*Lack of internship in organizations working on nutrition and food security like UNICEF, Word Food Program, FAO (GN01)* *No internship in research organizations or units specializing in nutrition. (GN10)* *The living conditions at POPOKABAKA were poor (GN11)* *Trip to Popokababka: a very bad road (GN12)* *The indifference of the administrative team faced with the difficulty encountered especially during internships (GN26)* *After the Popokabaka internship, I had the impression that the department had abandoned us [no communication on the rest of our programme] (GN17)*	*Improve: accessibility to Popokabaka, accommodation and catering conditions (GN05)* *To include internships in organizations interested in nutrition such as WFP, UNICEF, FAO,* etc.*, to enable learners to become familiar with different types of studies and approaches used by these organizations (GN09)* *Diversify internship sites on the last year of internship (GN11)* *I would like learners be trained in rural area and in united nations agencies (those working in nutritional fields) for increasing [the] chance for job [s] (GN19)* *If GROWNUT could contact some partners like WFP, FAO, etc, for some internship time.*
***Research experiences***		
*The workshop on research methodology (GN01)* *Mastering scientific research of nutritional problems, and data analysis software. (GN02)* *The [research] workshop in KwaZulu-Natal (GN17)* *Improving my research skills (GN18)* *Competence in research (GN19)* *Learning of research methods (GN23)* *Orientation towards to the research (GN27)* *Acquired the knowledge necessary to conduct research with ease (GN28)* *Improve my knowledge and skills in research (GN29)*	*Having two supervisors was a real challenge to face. (GN03)* *His [supervisor’s] support did not live up to my expectations. (GN05)* *Lack of understanding with my Bergen thesis supervisor (GN13)*	*Increase the practical work on research and data analysis with all software (Stata) (GN24)*

## Discussion

To address the lack of research and academic leadership skills in health and nutrition in LMICs, we describe a successful nutrition-focussed educational approach that could be adapted for other postgraduate programmes. In DRC, a very-low income country [16], this approach increased the number of nutrition researchers, significantly strengthening academic leadership and research skills, with wider benefits outside of the nutrition field. Skilled researchers drive evidence-based research agendas and lobby for policy changes to improve important health indicators. We suggest collaborative projects among higher education institutions provide feasible solutions to address the skills gaps identified in research and research output in LMICs [9, 14, 15].

This project was positioned as a three-way North-South-South partnership. Sustainability is a challenge for externally funded partnerships and our approach addressed this by increasing skills and confidence of local academic staff to take over delivery of the programme. Investing in KSPH academic staff has gone some way to promote sustainability, but the number of graduates has not reached critical mass yet. Partnerships are more likely to be successful and sustainable if maintained for periods longer than the normal five-year funding cycles. Longer funding periods would ensure ongoing support for novice programmes and create space for changes to occur, particularly in fluctuating academic landscapes where responses may be required to address changing disease patterns, epidemics and climate change. Achieving the skills to ensure sustainability requires long-term investment [23], and we contend there is still some way to go for KSPH to consolidate and build on what has been learned and achieved in this programme, whilst addressing challenges to effect transition from reliance on partner support to confidence, empowerment and independence.

Flexible training platforms [24], on-line learning methodologies [9] and blended learning approaches [13] are now more commonly used for teaching and learning as improvements to technology improves access for partners and students in LMICs. These approaches could address the high financial and human resource costs of extensive travel to and from partner countries, making North-South-South collaborations more cost effective. The set-up cost for on-line platforms may be high, but once established the platform will provide a range of opportunities to support teaching and learning and promote engagement with the scientific community, and in the longer term, may free resources for other important areas of educational programmes. Developing technology-based approaches that are supported by robust evidence of their quality and effectiveness is an important research need going forward.

In our study students were often constrained by financial and personal responsibilities, even those who received bursaries. Financial hardships, and even hunger, are not uncommon among university students [25–27], even in middle- and high-income countries, are therefore to be expected in a low-income country such as DRC, even among doctors and nutritionists who made up most of the student body. Despite being professionals, postgraduate students may have extensive financial and family responsibilities, making participation in post-graduate studies without financial support challenging. Funders often provide bursaries only for students designated as needy or based on merit, to avoid creating too much reliance on external funding, as in this project where funding was provided for a small number of students, and many applicants who did not receive a bursary withdrew as a result. Project funders should weigh the need to develop a critical mass of highly skilled qualified researchers against a reluctance to provide bursaries for a high proportion of students. We suggest that more bursaries at the start of the programme would allow it to become well established, and develop a large base of graduates to provide sustainability. The number of bursaries could then be reduced as the academic community becomes more able to sustain the process.

Most master’s students responded well to the experience of spending several months in a rural area, working with the community and gaining practical experience. Embedding students from an urban-based university in a rural research site for practical learning and research was a novel approach aligned with the authentic learning approach, and differed from other master’s programmes at KSPH or UNIKIN. Exposing or immersing medical students and other health professionals in rural areas has long been recommended as a way to recruit and retain health professionals in rural areas [28–30], and improve health worker coverage in remote areas [31]. Including a rural internship in a master’s programme has a strong rationale but also has a number of challenges that need to be addressed if it is to achieve maximum benefits. Learning during the rural internship needs to be well structured, students need to be supported and logistic constraints considered. Some students reported inadequate support during their internship and this distracted from their rural experience. Students are vulnerable in an unfamiliar environment and need extra support to establish themselves and benefit from the experience. The extremely poor roads to the rural research site prevented students from travelling home during their internship, which enhanced their isolation but also allowed them to experience rural life, which some students really appreciated. It is important when choosing a rural site to consider this balance and ensure that it is feasible for students to receive the required support from academic staff. Students suggested increasing the number of internship sites to include placements with national and international non-governmental organisations working in the nutrition field.

The use of English as the medium of instruction was a considered decision made by all partners, taking into consideration the wide-ranging benefits to the students and to the institution that have been observed in other settings [32]. Use of English was positively received and enhanced students’ ability to access literature and create research networks. However, a few students struggled with learning and writing in English, which may have detracted from their experience of the course and their ability to perform academically. We agree with students that pre-enrolment English courses and additional support in communication and writing in English should be included in similar programmes in future.

An important output of the programme was to strengthen research capacity, and while students completed their research projects and a few students presented their findings at conferences, no master’s student has thus far published their research. Writing articles for publication requires writing skills, dedicated efforts, time and on-going mentoring of young researchers but there also needs to be credible research to report. Future skills development programmes should provide enhanced support for writing for students and junior academic staff. In addition, data generated from studies did not lead to changes in nutrition policies. It is crucial to ensure that research findings are credible and communicated effectively to policy makers.

Nutrition challenges remain an ongoing concern in the DRC and it is unclear whether SDG 2 will be achieved. We propose that going forward a strong coherent nutrition research agenda based on local priorities is developed at KSPH, in partnership with PRONANUT, to which student research can contribute and which can explore solutions to the nutrition challenges affecting communities in DRC. Vibrant networks should be established both within the DRC and with other African countries findings disseminated to large and diverse audiences focussing applying findings to change policies.

### Strengths and weaknesses

The strength of this evaluation is the good response rate. Self-administered questionnaires reduced interviewer bias and were available in both French and English allowing participants to freely express themselves. The findings from this study are specific to the programme described and therefore do not represent students in other programmes.

## Conclusion

Early achievements and successes from this master’s programme renews our commitment to health research capacity in resource constrained countries and universities through development of highly qualified academics and good quality academic programmes. The authentic learning pedagogy provided students with critical skills needed to advocate for the nutrition agenda in the DRC and provides a model for creating a teaching platform focusing on nutrition and research. However, creating capacity takes time and long-term funding is needed to build on early success and ensure sustainability of partnerships in higher education. Blended learning approaches may provide more cost-effective teaching and learning methodologies within North-South partnerships, but more research is required to establish their effectiveness.

## Supplementary Information

**Additional file 1: Supplementary File** – Data collection tool.

Additional file 2

## Data Availability

Data supporting the findings of this study are available from the principle investigator Centre for Rural Health (email: horwoodc@ukzn.ac.za) or as attached supplementary files.

## Abbreviations

CIH: Centre for International Health
CRH: Centre for Rural Health
DRC: Democratic Republic of Congo
FELTP: Field Epidemiology and Laboratory Training Programme
GROWNUT: Growing Partnership for Higher Education and Research in Nutritional Epidemiology
HSSREC: Humanities and Social Sciences Research Ethics Committee
JICA: Japan International Cooperation Agency
KSPH: Kinshasa School of Public Health
LMIC: Low-Middle income countries
MPH: Masters in Public Health
NORHED: Norwegian Programme for Capacity Development in Higher Education and Research for Development
PRONANUT: Programme National de Nutrition (National Nutrition Programme / Ministry of Health, DRC)
SDG: Sustainable Development Goals
SCONUTAL: Societé Congolaise de Nutrition et d’Alimentation
UKZN: University of KwaZulu-Natal
UNIKIN: University of Kinshasa
UNICEF: United Nations International Child Emergency Fund
WFP: World Food Programme
WHO: World Health Organisation
