# Supplementary material for: Establishing a postgraduate programme in nutritional epidemiology to strengthen resource capacity, academic leadership and research in the democratic republic of Congo
Source: BMC Med Educ. 2021 Feb 27;21:136. doi: 10.1186/s12909-021-02557-3 (PMC7912884; doi:10.1186/s12909-021-02557-3)
Supplement: Supplementary file 1 — Additional file 1: Supplementary File – Data collection tool. [file 12909_2021_2557_MOESM1_ESM.docx]

**SUPPLEMENTARY FILE – DATA COLLECTION TOOL**

**ESTABLISHING A POSTGRADUATE PROGRAMME IN NUTRITIONAL EPIDEMIOLOGY TO STRENGTHEN RESOURCE CAPACITY, ACADEMIC LEADERSHIP AND RESEARCH IN THE DEMOCRATIC REPUBLIC OF CONGO**

Mapatano Mala Ali^1^, Lyn Haskins^2^, Vaughn John^3^, Anne Hatløy ^4.5^, Silondile Luthuli^2^, Sphindile Mapumulo^2^, Ingunn M.S. Engebretsen^4^, Thorkild Tylleskär^4^, Paulin Mutombo^1^, Christiane Horwood^2^

**Affiliations**

^1^Kinshasa School of Public Health, University of Kinshasa, Democratic Republic of Congo

^2^Centre for Rural Health, School of Nursing and Public Health, University of KwaZulu-Natal, Durban, South Africa

^3^School of Education, University of KwaZulu-Natal, Pietermaritzburg, South Africa

^4^Centre for International Health, University of Bergen, Norway

^5^Fafo Institute for Labour and Social Research, Oslo, Norway

**Corresponding author:**

Lyn Haskins

haskins@ukzn.ac.za

**ESTABLISHING A POSTGRADUATE PROGRAMME IN NUTRITIONAL EPIDEMIOLOGY TO STRENGTHEN RESOURCE CAPACITY, ACADEMIC LEADERSHIP AND RESEARCH IN THE DEMOCRATIC REPUBLIC OF CONGO**

DATA COLLECTION TOOL – SELF ADMINISTERED ON-LINE

| **GROWNUT evaluation survey: participant information** | | | |
| --- | --- | --- | --- |
|  | Participant number (study generated) |  | |
|  | GROWNUT cohort number (write 1-4) |  | |
|  | Age (write age in years) |  | |
|  | Gender | Male | Female |
| 4. | Profession/basic degree |  | |
|  | Did you participate in an internship in Popokabaka? | Yes | No |
| 5. | Was the data for your thesis collected in Popokabaka? | Yes | No |
| 6. | Degree complete | Yes | No |
| 7. | Did you receive a GROWNUT bursary? | Yes | No |
| 8. | Did you have a co-supervisor from a GROWNUT partner university? | Yes | No |
| 9. | If yes, from which University was your co-supervisor? | University of Bergen, Norway | University of KZN,  South Africa |
| 10. | Did you write your thesis in English or in French? | English | French |

| Read each statement and rate the quality of each activity according to your experience by ticking in the box very poor/poor or inadequate/ neutral/ good/ very good. If activities are mentioned that you did not participate in or were not available to your cohort tick not applicable. Please be honest as this will help us to improve the GROWNUT nutritional epidemiology Masters programme for future students. **Your information will be kept confidential and no information will be linked to any individual student at any time.** | | | | | | |
| --- | --- | --- | --- | --- | --- | --- |
|  | **Very poor** | **Poor** | **Neutral** | **Good** | **Very good/Excellent** | **Not applicable/was not exposed** |
| **RATE THE QUALITY OF THE ACTIVITY** | **1** | **2** | **3** | **4** | **5** |  |
| Selection process for entry to the GROWNUT masters course |  |  |  |  |  |  |
| **Curriculum: teaching modules** |  |  |  |  |  |  |
| Content of the curriculum |  |  |  |  |  |  |
| Teaching approach/method used |  |  |  |  |  |  |
| Formal teaching modules conducted at KSPH |  |  |  |  |  |  |
| Formal teaching modules conducted at UKZN |  |  |  |  |  |  |
| External facilitators from GROWNUT partners |  |  |  |  |  |  |
| Access to library facilities (physical and electronic) at KSPH |  |  |  |  |  |  |
| **Internship in Popokabaka (rural research site)** |  |  |  |  |  |  |
| Teaching provided during the internship in Popokabaka |  |  |  |  |  |  |
| Skills development activities in Popokabaka |  |  |  |  |  |  |
| Accommodation provided in Popokabaka |  |  |  |  |  |  |
| Access to library facilities (physical and electronic) in Popokabaka |  |  |  |  |  |  |
| Support provided during activities in Popokabaka |  |  |  |  |  |  |
| Interactions with the community in Popokabaka |  |  |  |  |  |  |
| **Supervision of research/ thesis writing** |  |  |  |  |  |  |
| Support provided in developing your research proposal |  |  |  |  |  |  |
| Support provided while collecting your research data |  |  |  |  |  |  |
| Support & feedback provided during thesis writing |  |  |  |  |  |  |
| Support provided by your KSPH supervisor |  |  |  |  |  |  |
| Support provided by your co-supervisor (from Bergen or UKZN) |  |  |  |  |  |  |
| **Read the statements below and respond whether you agree or disagree with the statement** | | | | | | |
|  | **Strongly disagree** | **Disagree** | **Neutral** | **Agree** | **Strongly agree** | **Not applicable** |
| The learning approach used in GROWNUT allowed a high level of student participation |  |  |  |  |  |  |
| I really benefitted from learning and writing in English |  |  |  |  |  |  |
| It was a problem having two supervisors I would have preferred to have only one supervisor |  |  |  |  |  |  |
| I aim to make my career in the field of nutrition or nutritional epidemiology |  |  |  |  |  |  |
| I learned all the skills needed to conduct my research project before starting data collection |  |  |  |  |  |  |
| Collecting data in a rural area enhanced the learning experience for my Master degree |  |  |  |  |  |  |
| Facilitators from universities of Bergen and KwaZulu Natal did not understand the requirements of students in DRC |  |  |  |  |  |  |
| I received relevant and timely feedback from my supervisors |  |  |  |  |  |  |
| I have developed research skills that I will use in my future career |  |  |  |  |  |  |
| I was often not able to understand the topics discussed in class because I was not able to understand the English |  |  |  |  |  |  |
| I experienced personal or family challenges which affected my studies |  |  |  |  |  |  |
| The bursary I received was sufficient |  |  |  |  |  |  |
| The feedback I received from my two supervisors did not always agree |  |  |  |  |  |  |
| The challenge of writing in English prevented me from writing an excellent thesis |  |  |  |  |  |  |
| It was difficult to pay for my bills/expenses while I was studying |  |  |  |  |  |  |
| My study has improved my practice as a health practitioner |  |  |  |  |  |  |
| The best thing about studying for a GROWNUT Master degree in Nutritional epidemiology was: | | | | | | |
| The worst thing about studying for a GROWNUT Master degree in Nutritional epidemiology was: | | | | | | |
| Any other comments you would like to add | | | | | | |
| Describe your current position and how you are using your nutrition epidemiology skills in your day-to-day work | | | | | | |
